# Detection of active pharmaceutical ingredients in surface water polluted by an informal settlement

**DOI:** 10.1007/s10661-025-14636-9

**Published:** 2025-10-02

**Authors:** Kalpana Maraj, Emily Nicklin, Cesarina Edmonds-Smith, Kevin Winter

**Affiliations:** 1 Future Water Institute, University of Cape Town, Cape Town, 7701 South Africa; 2 Department of Chemistry, University of Cape Town, Cape Town, 7701 South Africa; 3 Department of Chemical Engineering, University of Cape Town, Cape Town, 7701 South Africa; 4 Department of Environmental and Geographical Sciences, University of Cape Town, Cape Town, 7701 South Africa

**Keywords:** Emerging contaminants, Global South, Peri-urban catchments, Surface water quality, Temporal water quality monitoring

## Abstract

The presence of active pharmaceutical ingredients (APIs) and other pollutants in rivers receiving runoff from informal settlements remains poorly understood, particularly in peri-urban environments with limited wastewater treatment infrastructure. This study aimed to monitor the presence of APIs and other pollutants in the Stiebeuel River, which receives polluted runoff from an informal settlement (estimated population of 8100) in the Western Cape, South Africa. A discrete automatic sampler was used to capture temporal variations in pollutant concentrations over 24 h in April and May 2024. The study was conducted during the dry season due to the reduced dilution effect of rainfall and runoff. Samples were analysed for physical and chemical parameters, including ammonia, orthophosphate, sulphate, total organic carbon, and *E. coli*, as well as 14 targeted APIs and lifestyle markers using ultra-performance liquid chromatography–mass spectrometry. The results indicate elevated concentrations of nutrients and *E. coli*, with peak pollutant loads occurring between 10:00 and 16:00, most likely associated with heightened human activity in the settlement. API analysis confirmed the presence of 11 compounds from a targeted list of 14, including antiretrovirals (efavirenz, nevirapine, ritonavir), antibiotics (trimethoprim), and analgesics (paracetamol, diclofenac), among others. These findings suggest that informal settlements discharge elevated concentrations of nutrients and act as a source of API pollution with potential downstream impacts for users and aquatic ecosystems. The findings highlight the need for improved water quality monitoring in South African catchments impacted by informal settlement runoff.

## Introduction

### Background

Active pharmaceutical contaminants (APIs) have been detected in various water bodies, including surface water, groundwater, stormwater and wastewater influents and effluents (Ngqwala & Muchesa, 2020; Waleng & Nomngongo, 2022; Wilkinson et al., 2022). Many of these APIs are of concern due to their potential adverse effects on environmental and human health, such as contributing to antimicrobial resistance or causing endocrine-disrupting effects (Kunene & Mahlambi, 2023; Souza et al., 2022; Su et al., 2020; Swartz et al., 2018; Tang et al., 2020). These contaminants primarily originate from wastewater treatment plants (WWTPs), which are largely ineffective at removing APIs from wastewater, as evidenced by the detection of both parent drugs and their degradation products in receiving surface waters (Funke et al., 2016; Archer et al., 2020; Munzhelele et al., 2024; Netshithothole et al., 2024). Other notable API sources include landfill leachate, effluent from hospitals or medical research facilities, and agricultural runoff (Adeola & Forbes, 2022).

Poorly serviced informal settlements are potential sources for API pollution due to inadequate access to centralised sanitation, forcing residents to rely on on-site sanitation systems such as pit latrines or open defecation (Horn et al., 2022). In addition, the lack of proper waste disposal facilities in these areas often leads to the indiscriminate disposal of pharmaceuticals, which can ultimately contaminate the environment. These waste streams, consisting primarily of greywater and “blackwater” (i.e. sewage), contribute to other water quality challenges, including elevated levels of nutrients, organic pollutants and microbiological pollutants (Carden et al., 2007).

Several studies in sub-Saharan Africa have investigated the impact of informal settlements on surface water quality. These primarily include in-situ measurements of physicochemical indicators (e.g., pH, dissolved oxygen, temperature, and conductivity) as well as more in-depth analyses of nutrient and organic loadings and faecal coliforms (Fatoki et al., 2001; Edokpayi et al., 2015; Gqomfa et al., 2022; Morole et al., 2023; Gwenzi et al., 2024). More recently, research has shifted toward analysing contaminants of emerging concern (CECs) in surface waters affected by informal settlements, detecting various APIs, including antibiotics and antiretroviral drugs (ARVs), among others (Chebii et al., 2024; Holton et al., 2022; Horn et al., 2022; Kairigo et al., 2020).

Ngumba et al. (2016) reported surface water concentrations of antibiotics such as ciprofloxacin and sulfamethoxazole in the Nairobi River Basin ranging from 2.5 to 32.6 μg/L, consistently higher than those detected in effluent from domestic wastewater treatment plants, which were typically below 5 μg/L. Similarly, Chebii et al. (2024) found that analgesics, anti-inflammatory drugs, ARVs, and antibiotics were prevalent in rivers downstream of informal settlements in the Athi River Basin, with efavirenz concentrations reaching up to 100 μg/L, and lamivudine between 0.01 and 0.1 μg/L.

In South Africa, over 100 different types of APIs have been detected in various water bodies across the country (Madikizela, 2023). These include antibiotics, analgesics, anti-inflammatories, hormones, non-steroidal anti-inflammatory drugs, beta-blockers, blood lipid regulators and anti-epileptics, occurring at varying concentrations depending on the location. While most studies identify WWTP effluent as the primary source of API pollution, growing evidence suggests that informal settlements also contribute significantly to API pollution. Horn et al. (2022) identified undocumented waste discharges and sewer outfalls from informal settlements in South Africa as primary sources of antiretroviral contamination, with efavirenz concentrations ranging from 0.8 to 38.5 μg/L. Additionally, reported concentrations in receiving waters influenced by both WWTP discharge and informal settlement runoff include efavirenz (0.1–100 μg/L), lamivudine (0.01–228 μg/L), nevirapine (0.018–10 μg/L), and emtricitabine (0.0005–226 μg/L), among others (Chebii et al., 2024; Holton et al., 2022; Horn et al., 2022; Kairigo et al., 2020). Surface water samples were collected downstream of multiple potential pollution sources in these studies, including WWTPs, informal settlements and landfill runoff, making it difficult to isolate the contribution of any single source of pollution. These findings highlight the importance of understanding the role of informal settlements in surface water contamination, particularly in relation to pharmaceutical pollution.

While previous studies have made significant contributions to our understanding of the potential impact of informal settlements on API pollution in surface waters, there are limitations in the sampling approaches used to quantify these impacts. First, many of these studies were conducted in catchments with multiple competing land uses, such as major WWTPs, which could potentially contribute to API pollution, making it difficult to distinguish the specific impacts of informal settlements (Archer et al., 2023; Chebii et al., 2024; Horn et al., 2022). Second, these studies largely relied on grab sampling over a pre-determined period of time, capturing only a snapshot of water quality at a single point in time (Chebii et al., 2024; Horn et al., 2022; K’oreje et al., 2016; Matongo et al., 2015; Waleng & Nomngongo, 2022; Wilkinson et al., 2022). As a result, they fail to account for the daily fluctuations in water quality and APIs, leading to a limited understanding of peak pollutant periods and variations in the types of APIs present throughout the day.

These sampling limitations can largely be attributed to the complex challenges of conducting research in informal settlements. Beyond the infrastructural and socio-economic conditions that contribute to poor service delivery and compromised water quality, informal settlements often experience elevated levels of crime. This is frequently linked to broader issues of poverty and unemployment. As a result, ensuring the security of monitoring equipment, particularly for continuous sampling, remains a significant challenge (Pereira & Xavier Junior, 2020). As a result, monitoring regimes are often limited to grab samples collected at specific times of the day when it is deemed safe for the researcher (Sheridan et al., 2023). However, this approach provides only limited insight into the dynamic conditions of informal settlements, which are largely driven by human activity.

Capturing these dynamic conditions through a temporal sampling regime and determining the extent to which informal settlements contribute to API pollution and other key contaminants are therefore key research gaps addressed by this study. This study aimed to establish a sampling regime capable of capturing daily fluctuations in water quality, with a focus on the qualitative detection of APIs and lifestyle markers in a river receiving runoff from an informal settlement. The research was conducted at the Water Hub, a research and innovation centre, situated at a secure site alongside a river downstream from an informal settlement in a peri-urban catchment in Franschhoek (Western Cape, South Africa). The study aims to provide further insight into daily fluctuations and peak periods of pollutant presence, helping to identify when elevated pollutant concentrations occur in the river system. While direct health risks were not assessed, understanding these patterns can inform water reuse practices and support the development of strategies to mitigate peak pollutant loads.

## Materials and methods

### Study area

**Fig. 1:**
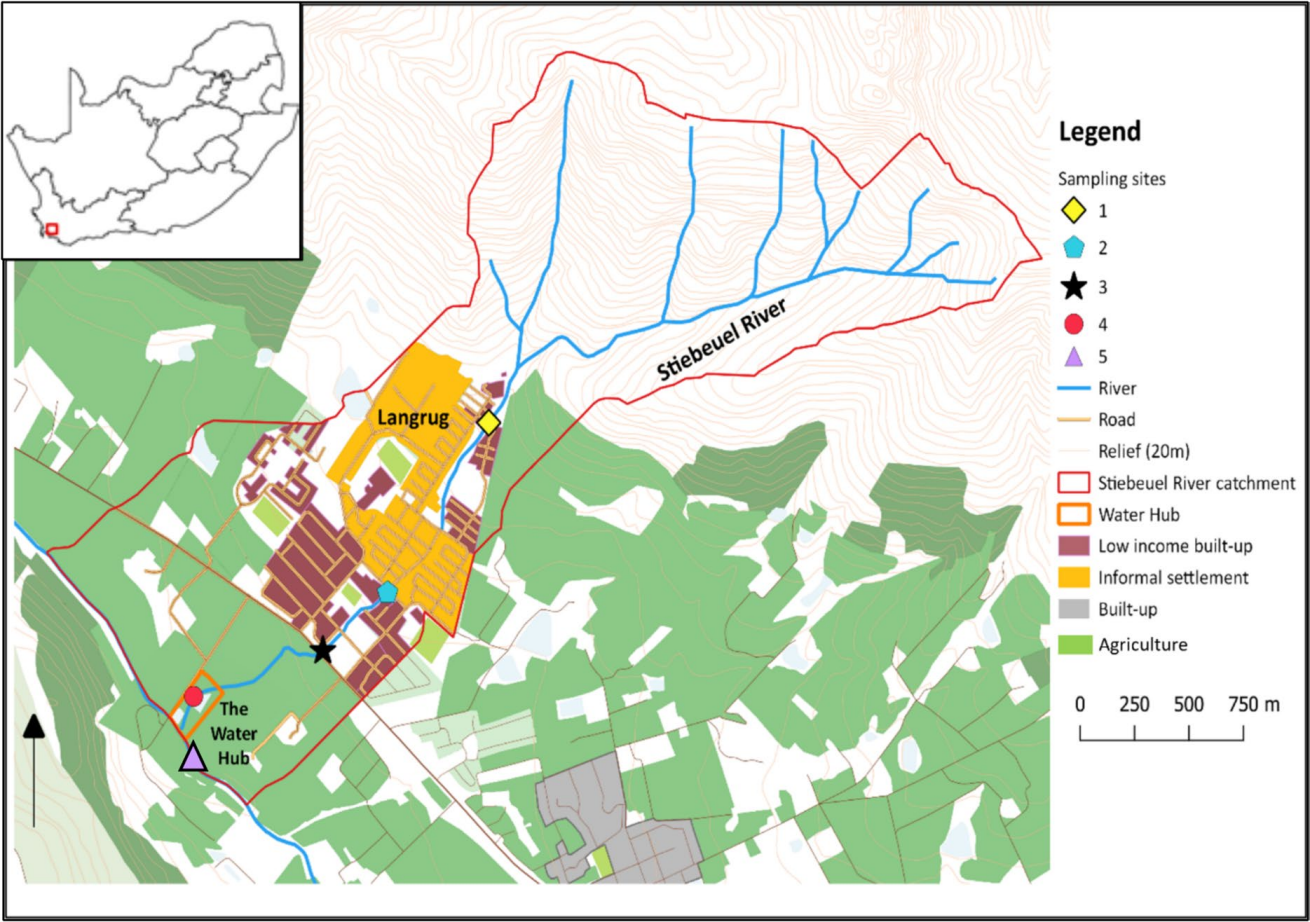
the Stiebeuel River catchment and sampling sites located along the river

The study was conducted at the Water Hub, which is a research site and living lab located close to the formal town of Franschhoek in the Western Cape province of South Africa. A map of the area is shown in Fig. 1. The Franschhoek Valley has a Mediterranean climate, with warm, dry summers and cool, wet winters. Rainfall is highly seasonal, with approximately 80% of precipitation occurring between April and September (Fell, 2017). Annual rainfall varies from 784 mm in the lower valley to 903 mm in the upper watershed, with peak rainfall differences of up to 133 mm between the dry and wet months. The average temperature is 16.4 °C, although extreme heat waves can drive temperatures to 45 °C between October and March (Winter et al., 2023).

The site is situated on the banks of the Stiebeuel River, which traverses a sub-catchment area of 4.37 km^2^ and flows into the Berg River, one of the primary watercourses in the region (Fell, 2017). The Stiebeuel River is a perennial stream with its source in the Hawequas Mountains, primarily fed by springs, rainfall, and surface runoff from both formal and informal settlements, along with discarded greywater and effluent from malfunctioning sanitation systems. The river experiences low flow periods during the dry season, while flash flooding can occur during heavy winter rains. Its morphology is characterised by a steep 1:12 gradient as it descends from the Hawequas Mountains, and its riverbed consists of loamy sand and clayey soils, influencing hydrological response and sediment transport in the system (Rebelo & Esler, 2022). The catchment encompasses a mix of land uses, including agriculture, the Langrug informal settlement, and the low-income formal residential areas of Groendal and Mooiwater. Agriculture in the region is largely composed of vineyards and fruit farms, while Langrug and Groendal contribute to the wastewater runoff due to inadequate sanitation infrastructure (Rebelo & Esler, 2022; Winter et al., 2023).

Water quality in the Stiebeuel River is severely impacted by sewage, litter and domestic wastewater, with the primary sources of pollution originating from Langrug informal settlement. Dysfunctional drainage systems and the direct discharge of untreated wastewater have resulted in elevated organic pollution and nutrient loads (Stellenbosch Municipality, 2019) which negatively affect water quality, habitat integrity, and aquatic biodiversity (Department of Water Affairs & Forestry, 1996). The most recent available population data from a 2016 census places the number of residents in the sub-catchment at approximately 8,100 (Stellenbosch Municipality, 2019). Given that Langrug’s population has increased by an estimated 8.2% (Stellenbosch Municipality, 2023) and further encroachment of informal housing on the slopes of the Hawequas mountain, the associated wastewater discharge and pollution loads entering the Stiebeuel River have also likely intensified.

### Non-targeted analysis to determine the presence of CECs in the river

The study involved two distinct sampling approaches. The first sampling approach was a non-targeted analysis conducted on grab samples collected from five sampling sites along the Stiebeuel River to screen for the presence of APIs. The results from the non-targeted analysis informed the selection of target compounds and a suitable single location for subsequent structured temporal monitoring. The non-targeted analysis was followed by a targeted temporal monitoring phase at a single site (Site 4), where discrete water samples were collected every two hours over 24-h periods in April and May 2024 using an automatic sampler.

A preliminary non-targeted analysis to identify the presence of CECs in the river, including pharmaceuticals, and lifestyle markers like caffeine. Sites were selected near pollution point sources, such as stormwater outlet pipes (site 2) discharging contaminated runoff from the informal settlement, containing stormwater, greywater and occasionally sewage. A sampling site upstream of the informal settlement (Site 1) was initially included for analytical comparison however, subsequent analysis revealed poor water quality at this location as well, likely due to open defecation. The sampling sites are shown in Fig. 1.

A grab sampling approach was used for the non-targeted analysis with samples being collected in April 2023 under low-flow conditions in the river. Acid-cleaned 1-L Schott bottles were rinsed three times with river water and lowered to mid-depth for sample collection. Samples were refrigerated at < 4 °C, protected from UV light in a hard cooler box during transport, and frozen immediately upon arrival at the laboratory. Solid phase extraction (SPE) was performed within 48 h of sample collection, and the extracted cartridges were then couriered to the University of Cape Town’s Clinical Pharmacology PK Laboratory for immediate analysis. Two separate analytical methods were used for the detection of APIs and lifestyle markers in the grab samples. First, samples were analysed using a Waters Acquity UPLC with a Q-Tof mass spectrometer for the detection of anti-tuberculosis and ARV drugs. The method included the following analytes, grouped by therapeutic class: antiretrovirals (tenofovir, lamivudine, zidovudine, emtricitabine, dolutegravir, abacavir, darunavir, lopinavir, atazanavir, efavirenz, ritonavir); anti-tuberculosis drugs and associated metabolites (isoniazid, acetyl-isoniazid, pyrazinamide, ethambutol, rifampicin, des-rifampicin, rifabutin, des-rifabutin, rifapentine, des-rifapentine, pretomanid, delamanid, DM6705 [delamanid metabolite], bedaquiline, M2 [bedaquiline metabolite], clofazimine); and other antimicrobials (levofloxacin, moxifloxacin, linezolid).Second, for the broader screening of additional pharmaceuticals, including other prescription drugs and substances of abuse, samples were analysed using a high-resolution mass spectrometer. Detection was performed using MassLynx software in conjunction with a proprietary spectral library containing over 700 compounds.

### Sampling regime and rationale for temporal monitoring phase

An ISCO 3710 automatic sampler (manufactured by Teledyne ISCO) was used to collect water samples from the Stiebeuel River, approximately 2 km downstream of the Langrug informal settlement, over a 24-h period (at sampling site 4 shown in Fig. 1). The automatic sampler was programmed to collect individual 400 ml samples every two hours, from 02:00 to 24:00 on a Wednesday (12 samples over 24 h), over three weeks during the months of April and May 2024. These sampling intervals were sufficient to ensure even data distribution across regular time points throughout the day, effectively capturing temporal variations in water quality and the influence of upstream human activities. Although more frequent sampling would have provided a higher-resolution dataset on river water quality dynamics, the high cost of CEC analysis was the primary limiting factor. Sampling was consequently conducted on Wednesdays for practical convenience and was repeated on three occasions. However, these cannot be considered true replicates due to the high variability in the water quality of the Stiebeuel River. Wednesday was selected for sampling as it best reflected typical weekday patterns of human activity and pollutant discharge, based on pilot testing and prior observations of daily routines in informal settlements, which differ from formal areas due to high unemployment rates and variable schedules.

Another major limitation of the sampling regime was that samples were collected from only a single location in the Stiebeuel River, i.e. at the Water Hub research site (sampling site 4). This was necessary because the automatic sampler had to be placed in a secure location to prevent theft or vandalism. Although the sample location is approximately 2 km downstream from the informal settlement, suggesting some degree of dilution in pollutant concentrations and a lag effect between when pollutants enter the river and when they are detected (e.g., typically from stormwater outlets that convey waste streams from the Langrug informal settlement), there are no other significant pollution sources along the Stiebeuel River. Therefore, there is a reasonable degree of confidence that the water quality measured at the Water Hub represents a diluted version of the water quality upstream due to natural attenuation mechanisms and hydrological processes.

The study was intentionally conducted during the dry season when pollutant concentrations were highest due to the reduced dilution effect of rainfall and runoff. This was confirmed by a previous study on seasonal nutrient levels in the river, which found significantly higher concentrations of ammonia and orthophosphate in the summer when lower rainfall resulted in reduced river flow (Maraj et al., 2024). The study was therefore, designed to provide insight into a 'worst-case' scenario regarding potential water quality-related risks under higher pollutant load conditions.

### Analytical methods for API detection for temporal monitoring phase

#### Chemicals and materials

Acetonitrile (ACN) and methanol (MeOH) were supplied by Romil Ltd. (Waterbeach, Cambridge, GB). Formic acid (FA) and water were obtained from Sigma Aldrich (St. Louis, MO, USA). Efavirenz, emtricitabine, lamivudine, ritonavir, tenofovir, abacavir sulphate, and lopinavir from Merck and were all certified reference material (Cape Town, South Africa). All solvents were LC–MS grade.

#### Method for qualitative detection of APIs during temporal monitoring

This method follows a previously published protocol used for pharmaceutical compound detection in aquatic samples (Mosekiemang et al., 2021).

i.i. Sample preparation and extraction.

Samples were preconcentrated by solid phase extraction (SPE) using PRiME HLB cartridges (60 mg/3 mL, Oasis, Waters, Milford, USA). The SPE procedure entailed conditioning the cartridges with ACN followed by MeOH, equilibration with deionised water (pH adjusted to 7), sample loading (10 mL sample at ~ 1 mL/min), bed rinsing with pH adjusted water, drying by vacuum for approximately 10 min followed by elution with 5% formic acid in methanol. The eluate was dried under N_2_ and reconstituted in MeOH/water (3/7, v/v).

ii.ii. Instrumental analysis (LC–MS/MS).

A Waters Acquity UPLC system (Waters, Milford, USA) coupled with a Synapt G2 Q-TOF mass spectrometer equipped with an electrospray ionisation (ESI) source was used in this study. This instrumentation was used in the temporal monitoring study for targeted detection of selected APIs. The analysis focused on compound detection based on accurate mass and retention time relative to available reference standards, but no absolute or relative quantification was performed. Chromatographic separation was achieved on an ACQUITY UPLC BEH C18 column (1.7 µm, 2.1 × 100 mm), maintained at 45 °C. The injection volume was 5 µL. A gradient elution programme using solvent A (0.1% FA) and solvent B (ACN) was applied, with the system re-equilibrated prior to each injection.

The Q-TOF mass spectrometer was operated in low collision energy mode (4 V) with a mass range of 100–2000 m/z. Ultrapure argon was used as the collision-induced dissociation (CID) gas. The cone and capillary voltages were set at 15 V and 2.5 kV, respectively, and the source temperature was maintained at 120 °C. Nitrogen was used as the cone gas at a flow rate of 50 L/h. The instrument was externally calibrated using sodium formate, and leucine enkephalin (m/z 556.2771) was used as a lockspray reference mass throughout the analysis.

### Water quality measurement methods for temporal monitoring phase

#### Physical parameters

A Hanna Multiparameter HI98194 probe was used to measure pH, dissolved oxygen (DO), and electrical conductivity (EC) upon arrival at the laboratory. In-situ measurements were not feasible due to theft and vandalism risks. However, previous monitoring showed relatively stable pH (7.19–7.62), and EC remains valid within 28 days of collections if stored appropriately (Hach Company, 2012).

#### Ammonia, orthophosphate and sulphate

Ammonia, orthophosphate and sulphate were measured using a ThermoFisher Scientific Galley discrete analyser, which uses colorimetric and enzymatic measurements from a single sample through photometric analysis. After calibration, 10 mL of the sample was filtered using a 0.45 μm syringe filter and transferred into a cuvette, where it was added to an incubation chamber and analysed the concentrations of ammonia, orthophosphate and sulphate.

#### Total organic carbon

Total organic carbon (TOC) was measured using an AnalytikJena multi-N/C 3100 analyser (Analytik Jena, 2020). Unfiltered samples were mixed and transferred to foil-covered TOC vials. The instrument uses high-temperature catalytic oxidation (800 °C) with non-dispersive infrared (NDIR) detection. Inorganic carbon (TIC) was analysed separately, and TOC was calculated as TC minus TIC. All samples were analysed in triplicate.

#### Faecal indicator bacteria analysis

*Escherichia coli* (E. coli) was analysed using 3 M™ Petrifilm™ E. coli/Coliform Count Plates, incubated at 35 °C for 24 h. Colonies with blue colour and gas bubbles were counted as *E. coli* colonies. Although not a regulatory standard, this method has shown acceptable sensitivity and specificity for environmental monitoring and is comparable to ISO 9308–1 culture-based methods (Bird et al., 2020). Its limitations in detecting very low concentrations were noted and considered during result interpretation.

### Statistical analysis

To assess the effects of time and date on various water quality parameters, a two-way analysis of variance (ANOVA) without replication was conducted for parameters that met the assumption of normality. A Shapiro–Wilk test was performed on each parameter to assess this assumption. Parameters that met the assumption of normality (NH₃, SO₄^2^⁻, EC and pH) were analysed using a two-way ANOVA with time and date as the independent variables, and the respective water quality parameter as the dependent variable. This parametric analysis was used to determine whether the time of sampling and date significantly influenced measured parameters, while also considering their interaction effects. While in situ measurements of physico-chemical parameters are ideal, the use of autosamplers allowed for consistent, time-aligned sampling; thus, despite potential post-collection alterations in redox-sensitive parameters such as pH and DO, the statistical analysis remains valid for assessing relative diurnal trends.

For parameters that did not meet the assumption of normality (PO₄^3^⁻, TOC, E. coli, DO, turbidity and number of APIs detected), the non-parametric Kruskal–Wallis test was used to assess the same temporal relationships. All statistical analyses were conducted using R software, and a significance threshold of p < 0.05 was applied to determine statistical significance, with results interpreted accordingly. Statistically significant main effects and interactions were further analysed to assess temporal trends in water quality variations within the study site.

## Results and discussion

### Non-targeted analysis for API detection

A preliminary non-targeted analysis was conducted to identify APIs and lifestyle markers present in the Stiebeuel River, focusing on grab samples taken from sites near point-source pollutants such as stormwater outlet pipes discharging contaminated runoff from the informal settlement. A site upstream of the informal settlement was also included for comparison (see Fig. 1 for sampling site locations). The non-target analysis detected a range of CECs, including antiretroviral (ARV) drugs, tuberculosis (TB) medications, illicit substances, and lifestyle marker residues. Table 1 shows the results of the non-targeted analysis.

**Table 1:** APIs detected in various sampling locations as per non-target analysis results

Sampling Location	ARVs Detected	TB Drugs Detected	Other Drugs and Lifestyle Markers Detected
Site 1	Tenofovir, Lamivudine, Emtricitabine, Dolutegravir, Abacavir, Lopinavir, Atazanavir, Efavirenz, Ritonavir	Pyrazinamide, Ethambutol, Moxifloxacin	Caffeine, Methamphetamine, Methaqualone
Site 2	Tenofovir, Lamivudine, Emtricitabine, Dolutegravir, Abacavir, Darunavir, Lopinavir, Atazanavir, Efavirenz, Ritonavir	Pyrazinamide, Ethambutol	Caffeine, Methamphetamine, Methaqualone, O-Desmethyltramadol, Tramadol
Site 3	Tenofovir, Lamivudine, Emtricitabine, Dolutegravir, Abacavir, Darunavir, Lopinavir, Atazanavir, Efavirenz, Ritonavir	Pyrazinamide, Ethambutol	Caffeine, Methamphetamine, Methaqualone, O-Desmethyltramadol, Tramadol
Site 4	Tenofovir, Lamivudine, Emtricitabine, Dolutegravir, Abacavir, Darunavir, Lopinavir, Atazanavir, Efavirenz, Ritonavir	Acetyl-Isoniazid, Pyrazinamide, Ethambutol, Levofloxacin	Caffeine, Codeine, Methaqualone, O-Desmethyltramadol, Tramadol, Metformin
Site 5	Tenofovir, Lamivudine, Emtricitabine, Dolutegravir, Atazanavir, Efavirenz	Pyrazinamide	Caffeine, Methaqualone

The confirmation of the presence of APIs and other emerging contaminants in the Stiebeuel River was motivation for a more detailed investigation, considering other major surface water pollutant and temporal variations in water quality in addition to API detection over time

Site 1 which is at the upper section of the river displays significant contamination with 9 ARVs, 3 TB drugs and other substances such as caffeine, methamphetamine, and methaqualone being detected at the site. This reveals the extent of contamination caused by polluted runoff from the informal settlement, given that Langrug informal settlement has encroached on the upper section of the river and higher up the slopes of the Hawequas mountains. ARVs are the most represented class of API present in the Stiebeuel River with 6 ARVs being detected at sampling site 5 (downstream of the Stiebeuel River and in the Franschhoek River) and 10 ARVs being detected at sampling sites 2, 3, and 4. In addition to ARVs and TB drugs, other detected substances included caffeine, indicating wastewater contamination from domestic activities, and carbamazepine, a widely used anticonvulsant known for its persistence in aquatic environments (Aus der Beek et al., 2016; Madikizela et al., 2017; Wilkinson et al., 2022). The presence of methamphetamine and methaqualone suggests possible illicit drug contamination, raising further concerns about the unregulated disposal of wastewater from informal settlements.

### Stiebeuel river water quality characterisation

Water quality results from the automatic sampling campaign are summarised in Table 2. Statistical analyses were conducted to evaluate the influence of time and sampling date on selected parameters, as described in the Materials and Methods section. Table 2 presents the measured ranges of selected parameters and the outcomes of the associated statistical analyses (p < 0.05).

**Table 2:** Summary of the water quality parameters, measured ranges, and statistical analysis, with significance defined as *p* < 0.05

Water Quality Parameter	Measured Range	Time Effect (p-value)	Date Effect (p-value)	Significance
pH	7.33—7.98	0.0002	0.4140	Time Significant
Dissolved Oxygen	3.48–5.73 mg/L	< 0.0001	< 0.0001	Both Significant
Electrical Conductivity	99.0–466 µS/cm	0.2079	0.0164	Date Significant
Turbidity	3.43 −67.0 NTU	0.6240	< 0.0001	Date Significant
Ammonia	2.51–27.0 mg/L	< 0.0001	0.9090	Time Significant
Orthophosphate	0.04–2.73 mg/L	0.0100	0.0380	Both Significant
Sulphate	2.04–26.1 mg/L	< 0.0001	0.0018	Both Significant
Total Organic Carbon	8.77–78.4 mg/L	0.0400	0.0080	Both Significant
*E. coli*	20 000–1 270 000 cfu/100 ml	0.0530	0.0131	Both Significant
*Number of APIs*	-	< 0.0001	0.663	Time Significant

The statistical analyses revealed that time had a significant influence on most parameters, particularly ammonia, orthophosphate, sulphate, TOC, *E. coli* and number of APIs (p-values below 0.05). This suggests that these pollutants exhibit noticeable temporal variations that are likely influenced by changes in discharge patterns (linked to human activities in the informal settlement), microbial activity and hydrological conditions. The effect of date was significant for DO, EC, turbidity, orthophosphate, sulphate, TOC, *E. coli* and number of APIs.

**Fig. 2:**
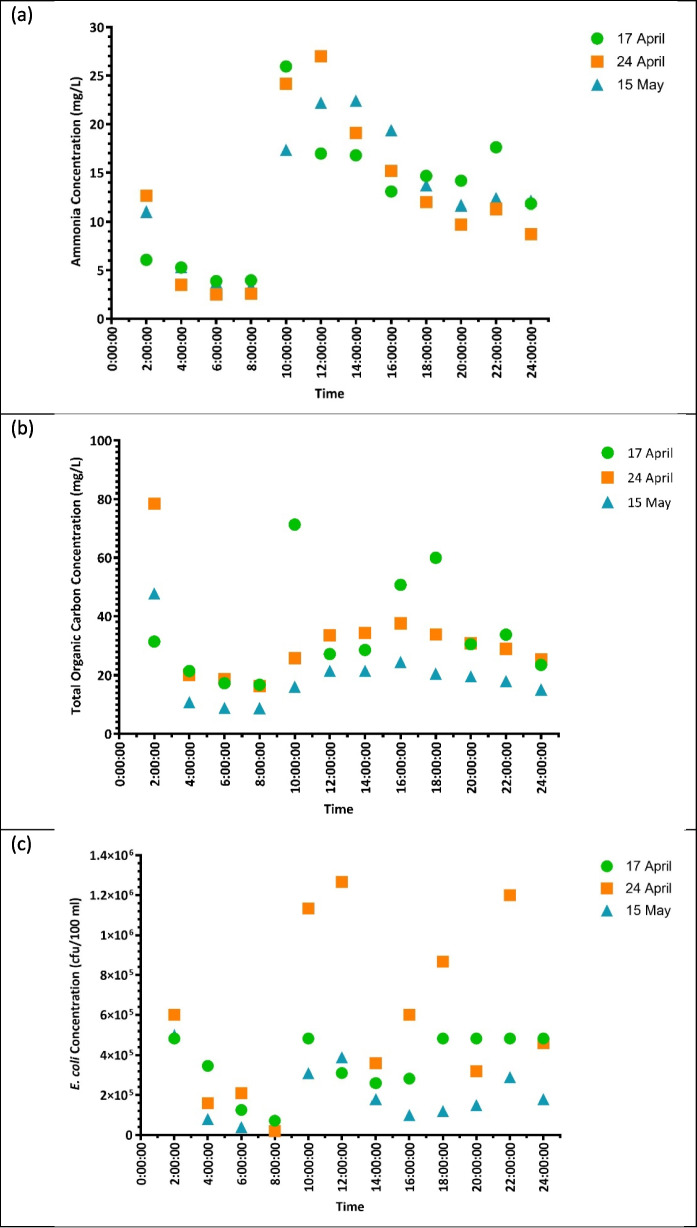
temporal variation in (**a)** ammonia concentrations, (**b)** total organic carbon concentrations and (**c**) E. coli levels over the study period

Figure 2 illustrates the temporal variations in three key water quality parameters (ammonia, total organic carbon and *E. coli*), showing fluctuations across different time points and sampling dates. These parameters have been selected to indicate pollution trends as they are an indicator of nutrient pollution, organic loading and microbial related human health risk. Ammonia levels varied significantly with time of day (p < 0.0001) but did not show significant variation across sampling dates. As shown in Fig. 2a, ammonia concentrations peaked between 10:00 and 16:00 before gradually declining in the evening hours. These peaks in concentration suggest episodic wastewater discharges, likely from domestic sewage or greywater entering the river. However, other factors such as temperature, pH, and microbial activity may also play a role in influencing ammonia levels in the river. The recorded ammonia concentrations far exceeded the recommended limit for aquatic ecosystems (≤ 18 μg/L) (Department of Water Affairs & Forestry, 1996), with a maximum value of 27 mg/L on 24 April.

TOC exhibited significant variations with both time (p = 0.0400) and date (p = 0.0080). As illustrated in Fig. 2b, TOC concentrations were highest between 10:00 and 16:00, consistent with ammonia concentration trends. Peaks in TOC suggest the presence of organic pollution sources, including greywater, sewage, and organic debris from the informal settlement. While no specific guideline exists for TOC, the observed high TOC concentrations (maximum of 78.4 mg/L measured on 24 April) indicate substantial organic pollution, likely originating from domestic sewage, greywater, and decaying organic material. Elevated TOC contributes to oxygen depletion in water bodies due to microbial decomposition, which can exacerbate hypoxic conditions and stress aquatic organisms (Gqomfa et al., 2022).

*E. coli* levels showed significant variations across both time (p = 0.0530) and date (p = 0.0131), indicating that both daily fluctuations and broader temporal trends influence microbial contamination. The highest E. coli concentrations were recorded on 24 April, with values exceeding 1 200 000 cfu/100 mL in some samples (Fig. 2c). The recorded E. coli levels in the Stiebeuel River reached a maximum of 1 200 000 cfu/100 mL, exceeding all South African water quality guidelines (< 1000 cfu/100 mL for irrigation and < 130 cfu/100 mL for recreation) (Department of Water Affairs & Forestry, 1996). This suggests severe faecal contamination, most likely from untreated sewage discharges and greywater runoff from the informal settlement. These values pose extreme risks for human and environmental health, particularly for downstream users relying on the river for recreation, irrigation, or domestic water use.

### Presence of APIs at sampling site 4 and comparison to other studies

**Fig. 3:**
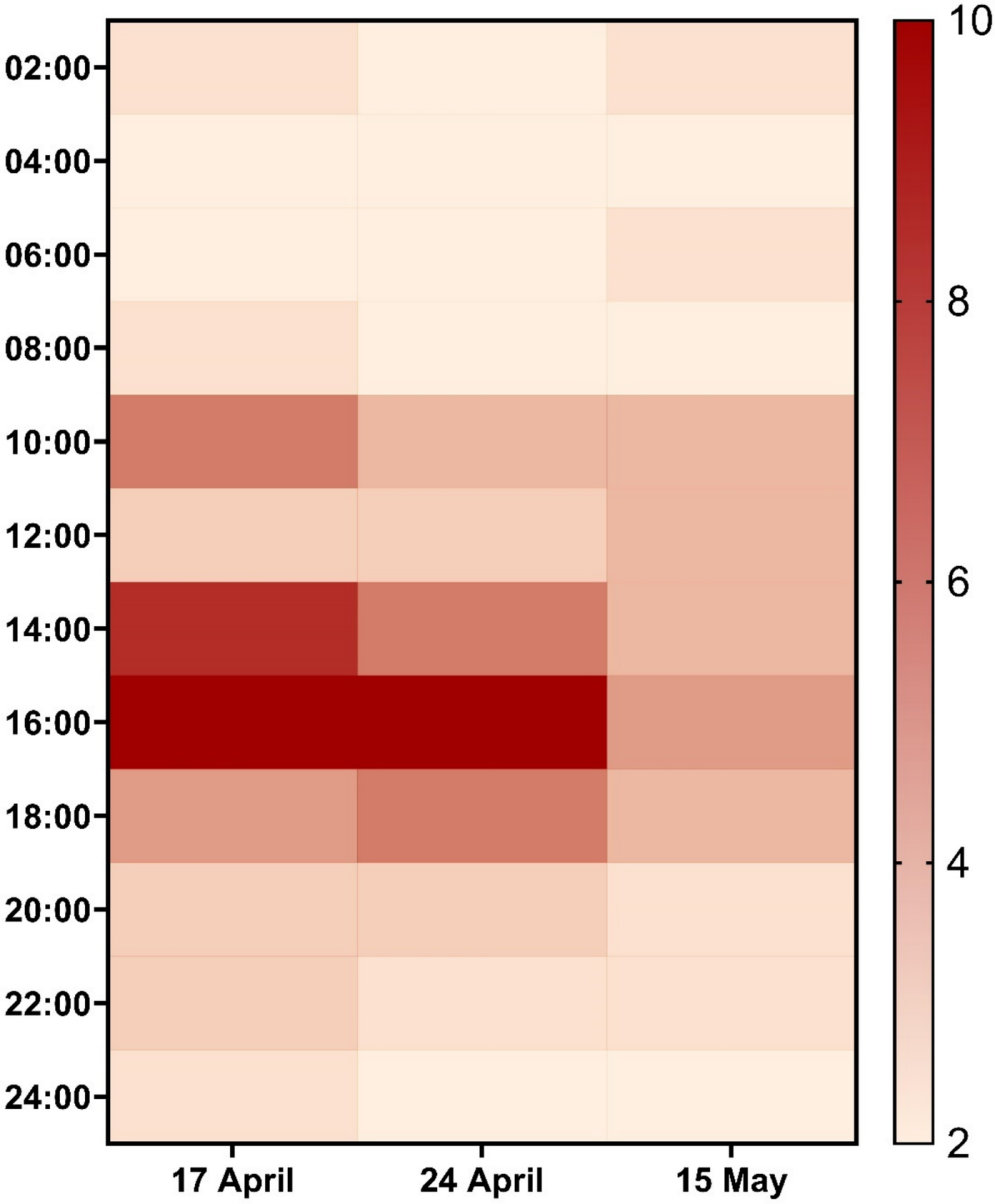
heatmap indicating the number of APIs detected over time during the study period

Various classes of pharmaceuticals were detected in the Stiebeuel River, including analgesics and anti-inflammatories (aspirin, paracetamol, diclofenac), antibiotics (trimethoprim), antiretrovirals (efavirenz, nevirapine, ritonavir, lopinavir), a central nervous system stimulant (caffeine), an anticonvulsant (carbamazepine), and a sedative-hypnotic (methaqualone). A full list of detected compounds and their comparison with other studies is provided in Table 3. The most abundant number of APIs were observed between 10:00 and 18:00 on each of the three days, as seen in Fig. 3. Mass spectral analysis identified the sedative methaqualone, a component of the illicit drug, Mandrax, as well as the antibiotic trimethoprim, anticonvulsant carbamazepine and caffeine. Anti-inflammatory drugs, including diclofenac, aspirin, and paracetamol, were confirmed through reference standards. ARVs detected through reference standards included efavirenz, lopinavir, ritonavir and nevirapine.

**Table 3:** Comparison between APIs detected in this study and other studies where informal settlements contribute to surface and groundwater pollution

Reference	Location	Water Source	APIs Identified
This Study	Stiebeuel River Catchment, Western Cape, South Africa	River	Aspirin, Caffeine, Carbamazepine, Diclofenac, Efavirenz, Lopinavir, Methaqualone, Nevirapine, Paracetamol, Ritonavir, Trimethoprim
Chebii et al. (2024)	River Athi Basin, Kenya – River Athi has 3 main tributaries which traverse Nairobi city and its densely populated informal settlement	River	Amantadine, Amitriptyline, Atenolol, Bezafibrate, Carbamazepine, Clarithromycin, Diazepam, Diclofenac, Efavirenz, Ibuprofen, Ifosfamide, Indomethacin, Ketoprofen, Lamivudine, Metoprolol, Metronidazole, Nalidixic Acid, Naproxen, Nevirapine, Oxytetracycline, Paracetamol, Propranolol, Salbutamol, Sotalol, Sulfadoxine, Sulfamethazine, Sulfamethoxazole, Tetracycline, Trimethoprim, Venlafaxine, Zidovudine
Twinomucunguzi et al. (2023)	Kampala, Uganda -Examined antibiotics in shallow groundwater sources underling urban informal settlements	Groundwater	Ampicillin, Benzylpenicillan, Chlortetracycline, Ciprofloxacin, Enrofloxacin, Metacycline, Nalidixic Acid, Oxytetracycline, Sulfathiazole, Tetracycline
Karimi et al. (2023)	Kisumu, Kenya -Examined antibiotics in groundwater sources underlying urban informal settlements	Groundwater	Sulfamethoxazole, Trimethoprim
Horn et al. (2022)	Multiple Rivers in the Gauteng Province, South Africa -Examined ARVs in rivers upstream and downstream from various WWTPs and informal settlements	River	Efavirenz, Lopinavir, Nevirapine, Ritonavir, Zidovudine
Ngumba et al. (2016)	Nairobi River Basin, Kenya -Examined antibiotics in Nairobi River Basin adjacent to informal settlements	River	Sulfamethoxazole, Trimethoprim, Ciprofloxacin

The pharmaceuticals identified in this study span multiple therapeutic classes with varying environmental persistence. Antiretrovirals such as efavirenz and ritonavir are widely prescribed in South Africa’s public healthcare system (approximately 5 million people receive antiretroviral therapy), and are excreted largely unmetabolised (Adeola & Forbes, 2022; Horn et al., 2022). These compounds are known to be resistant to natural attenuation processes and have been detected in other surface water bodies in similar contexts (Horn et al., 2022). Antibiotics such as trimethoprim, frequently used for co-infections and preventative treatments, are often persistent in aquatic environments and contribute to the development of antimicrobial resistance (Karimi et al., 2023). These compounds are often introduced into rivers via greywater discharge and tend to fluctuate with daily human activity. The presence of these APIs across multiple time points suggests sustained inputs and the limited dilution or degradation within the receiving water body during the dry season.

A comparison with similar studies conducted in regions where informal settlements contribute to surface and groundwater pollution indicates both commonalities and regional variations in the types of APIs detected (Table 3). While the comparison between this study and other investigations in similar contexts helps situate the findings within a broader research landscape, it is important to note several limitations. The detection of specific APIs is heavily influenced by the analytical methodologies employed in each study, including the sensitivity, selectivity, and scope of the targeted compounds. Differences in instrumentation, ionisation modes, spectral libraries, and sample preparation techniques can result in varying detection capabilities. Consequently, the absence of a particular API in one study does not necessarily indicate its true absence in the environment but may reflect limits of detection or differences in method performance. Moreover, the environmental and socio-economic pressures in each location vary significantly, such as population density, healthcare access, sanitation infrastructure, and patterns of pharmaceutical use, which can influence both the types and concentrations of APIs observed. These factors limit the direct comparability of absolute findings across studies, although qualitative similarities remain informative for identifying common pollution trends and shared challenges in informal settlement contexts.

The APIs identified in this study align with those reported in other South African and East African water systems, particularly in urban catchments influenced by WWTPs and informal settlements. The studies reported in Table 3 focusing on groundwater explicitly examined informal settlements as sources of pharmaceutical contamination. However, it is important to note that among the studies listed, this is the only investigation where the objective of the study was to assess an informal settlement as a primary source of pharmaceutical pollution in surface water. While previous surface water studies were conducted in larger river systems influenced by a variety of pollution sources, including WWTP effluents, this study focuses on a smaller peri-urban catchment with a more defined and localised pollution profile. The limited scale and singular upstream pollution source in this setting allowed for more focused observation of pollution arising from untreated greywater and sewage runoff from an informal settlement.

Studies conducted in the River Athi Basin, Kenya (Chebii et al., 2024) and the Nairobi River Basin (Ngumba et al., 2016) identified a broader range of pharmaceuticals, including beta-blockers, antidepressants, and additional antibiotics, some of which were not detected in the present study. The presence of these compounds suggests a higher diversity of pharmaceutical inputs in these systems, potentially linked to different prescription trends, healthcare infrastructure, and wastewater treatment efficiencies (Archer et al., 2020). The occurrence of antibiotics in shallow groundwater systems in Uganda and Kenya (Karimi et al., 2023; Twinomucunguzi et al., 2023) differs from the findings in surface waters, indicating possible variations in pharmaceutical transport, degradation, and adsorption in groundwater environments compared to riverine systems (Waleng & Nomngongo, 2022).

The presence of ARVs in the Stiebeuel River catchment (particularly at sampling site 4, which is located 2 km downstream of the informal settlement) is consistent with findings from Gauteng province, South Africa (Horn et al., 2022) where similar ARVs were detected in rivers upstream and downstream of WWTPs and informal settlements. APIs in surface water reflect ongoing challenges associated with untreated wastewater discharges, particularly in areas lacking adequate sanitation infrastructure. Differences in API composition among studies may be attributed to variations in pharmaceutical use, sampling site location along the course of the river, wastewater management practices, and hydrogeological factors influencing contaminant fate and transport.

## Implications on future research

This study provides novel insights into the occurrence and temporal variability of APIs in a river receiving runoff from an informal settlement. The findings confirm that informal settlements, which lack adequate wastewater management infrastructure, act as hotspots for API pollution in surface water systems. While previous research has focused on WWTP effluents as primary sources of APIs in aquatic environments, this study highlights an underexplored pollution pathway: the direct discharge of untreated greywater and contaminated runoff from informal settlements into natural water bodies. The diurnal variation in API detection suggests that human activity patterns strongly influence contaminant inputs into the river.

Future research should expand the spatial focus of API monitoring and should include absolute quantification of API concentrations to assess how these contaminants behave along the course of the river. By investigating the presence, persistence, and mobility of APIs at multiple locations, it will be possible to determine how hydrological processes, dilution, and natural attenuation mechanisms influence contaminant distribution. Such studies will provide a more complete picture of API fate in peri-urban river systems, distinguishing between localised contamination from direct runoff and broader pollution trends affecting downstream water users and aquatic ecosystems. Understanding these dynamics is essential for informing water quality management strategies in regions where urban expansion and inadequate sanitation contribute to the increasing pollution of surface waters.

## Conclusion

This study demonstrates that runoff from informal settlements contributes to the contamination of surface waters with active pharmaceutical ingredients (APIs). Analysis of water samples from the Stiebeuel River catchment revealed elevated concentrations of ammonia, TOC, and *E. coli*, exceeding South African water quality guidelines. The presence of multiple pharmaceuticals, including antiretroviral drugs, antibiotics, and analgesics, confirms that informal settlements act as sources of API pollution. The study also identified diurnal variations in pollutant occurrence, with the greatest detection of APIs between 10:00 and 16:00, likely linked to human activity patterns. These findings expand current knowledge on API contamination in peri-urban river systems in the Global South and reinforce the need to consider informal settlements as key contributors to pharmaceutical pollution in surface waters. While the study is limited by its focus on a single catchment and constrained temporal sampling periods, these limitations mean the work is best understood as an exploratory case study that highlights key patterns and knowledge gaps, rather than a definitive quantification of informal settlement contributions. Nonetheless, by demonstrating that APIs can persist beyond the influence of traditional wastewater treatment plant discharge points, this research offers new evidence supporting the need for improved pollution and waste management in rapidly urbanising regions.

## Data Availability

All raw data associated with this study is available at the following link: [10.25375/uct.28523828.v1](https:/doi.org/10.25375/uct.28523828.v1).
